# Spasms after spinal cord injury show low-frequency intermuscular coherence

**DOI:** 10.1152/jn.00112.2018

**Published:** 2018-08-01

**Authors:** Stefane A. Aguiar, Stuart N. Baker, Katie Gant, Jorge Bohorquez, Christine K. Thomas

**Affiliations:** ^1^Institute of Neuroscience, Newcastle University, Newcastle Upon Tyne, United Kingdom; ^2^The Miami Project to Cure Paralysis, University of Miami Miller School of Medicine, Miami, Florida; ^3^Department of Biomedical Engineering, University of Miami Miller School of Medicine, Miami, Florida; ^4^Department of Neurological Surgery, University of Miami Miller School of Medicine, Miami, Florida; ^5^Department of Physiology and Biophysics, University of Miami Miller School of Medicine, Miami, Florida

**Keywords:** EMG-EMG coherence, spasms, spinal circuitry, spinal cord, spinal cord injury

## Abstract

Intermuscular coherence allows the investigation of common input to muscle groups. Although beta-band (15–30 Hz) intermuscular coherence is well understood as originating from the cortex, the source of intermuscular coherence at lower frequencies is still unclear. We used a wearable device that recorded electromyographic (EMG) signals during a 24-h period in four lower limb muscles of seven spinal cord injury patients (American Spinal Cord Injury Association impairment scale: A, 6 subjects; B, 1 subject) while they went about their normal daily life activities. We detected natural spasms occurring during these long-lasting recordings and calculated intermuscular coherence between all six possible combinations of muscle pairs. There was significant intermuscular coherence at low frequencies, between 2 and 13 Hz. The most likely source for this was the spinal cord and its peripheral feedback loops, because the spinal lesions in these patients had interrupted connections to supraspinal structures. This is the first report to demonstrate that the spinal cord is capable of producing low-frequency intermuscular coherence with severely reduced or abolished descending drive.

**NEW & NOTEWORTHY** This is the first report to demonstrate that intermuscular coherence between lower limb muscles at low frequencies can be produced by the spinal cord with severely reduced or abolished descending drive.

## INTRODUCTION

Intermuscular coherence is an important and useful tool in motor control studies. It can be used to investigate muscle groups receiving common input from parts of the nervous system (e.g., Nazarpour et al. 2012), to assist in the diagnosis of postural tremor (van der Stouwe et al. 2015) and upper motor neuron dysfunction in motor neuron disease (Fisher et al. 2012), and, when combined with corticomuscular coherence, to differentiate between pathways converging onto spinal motoneurons (Boonstra 2013). Because intermuscular coherence requires only the use of surface electromyography (EMG) recordings, it can be measured straightforwardly and noninvasively in humans. Analysis of coherence detected at different frequencies provides important information on how the nervous system works to control muscle activity during different tasks.

Intermuscular coherence in the beta band (15–30 Hz) is accepted to have a cortical origin. Corticomuscular coherence can also be observed in this frequency band (e.g., Baker et al. 1999; Conway et al. 1995); the common cortical drive to multiple muscles leads to intermuscular coherence (e.g., Kilner et al. 1999; Power et al. 2006). Beta oscillations are carried down the corticospinal tract (Baker et al. 2003), and damage to this pathway leads to loss of beta-band intermuscular coherence (Fisher et al. 2012). This may have substantial clinical relevance to assist in the early diagnosis of upper motor neuron dysfunction in motor neuron disease.

Although intermuscular coherence in the beta band is well understood, the mechanisms and origin of intermuscular coherence at lower frequencies are still under debate. Boonstra and colleagues (2009b) argue for a noncortical origin for EMG synchronization around 10 Hz, which has been observed on a number of different motor tasks in healthy humans (e.g., Boonstra et al. 2007; Evans and Baker 2003; Halliday et al. 2003). The typical lack of corticomuscular coherence around this frequency provides support for this argument (Boonstra et al. 2009b); indeed, it has been proposed that specific spinal circuits act to minimize cortico-muscular coupling in this band (Koželj and Baker 2014; Williams and Baker 2009; Williams et al. 2010). The variable function of such circuits may explain why ~10 Hz cortico-muscular coherence can occasionally be seen (Raethjen et al. 2002; Williams et al. 2009). In incomplete spinal cord injury, Bravo-Esteban et al. (2014, 2017) demonstrated that cortico-muscular coherence around 10–16 Hz correlated with the degree of spasticity. It remains to be determined which subcortical neural structure generates low-frequency rhythmic activity. One possibility is the brain stem (e.g., Boonstra et al. 2009b; Grosse and Brown 2003), because invasive recordings from the reticular formation in monkey show clear synchronization with peripheral oscillations in this band (Williams et al. 2010).

After spinal cord injury, patients commonly experience involuntary muscle spasms; these can affect patient’s lives to varying degrees (Thomas et al. 2014b). Spasms can be classified into different types, including tonic EMG activity, clonus, and unit firing (Winslow et al. 2015). After spinal cord injury, the contribution of afferent input to motoneuron output may increase: in some cases, a spasm can easily be evoked by stimuli such as light touch and small postural adjustments. Other contributors to spasm generation may be changes in intrinsic motoneuron properties, such as a reduction in the spiking threshold, and a reduction in postsynaptic inhibition (Thomas et al. 2014a).

Recent advances in technology have allowed long-term recording of EMG activity outside the laboratory (Brown et al. 2016; Zaaimi et al. 2018). Such recordings have allowed quantitative characterization of types and incidence of spasms after spinal cord injury (Thomas et al. 2014b), providing data on many more instances of spasms than is possible in a brief laboratory visit. In this report, we use such recordings to explore shared drive to muscles using intermuscular coherence. We find clear evidence for coupling at 2–13 Hz. Because these patients had spinal lesions that severely reduced or abolished descending drive, it is likely that spinal or afferent circuits below the level of the lesion generate this rhythmic activity.

## MATERIALS AND METHODS

This report uses EMG data collected as part of a previous study that developed methods for detection and classification of spasms (Winslow et al. 2015). Seven spinal cord injury volunteers, 28 to 66 yr old (5 men, 2 women) took part in the study. Level and time of lesion varied from C3 to C7 and from 5 to 28 yr, respectively. The American Spinal Cord Injury Association Impairment (ASIA) scale was A (*n* = 6) or B (*n* = 1). An ASIA rating of A indicates a lack of motor or sensory function below the level of the lesion. ASIA B also indicates a complete motor deficit, although there is some retained sensory function. A lack of motor function on the ASIA scale is assessed by the failure to generate a visible or palpable voluntary contraction of a limb segment. All participants signed a written consent form, and all procedures were approved by the University of Miami Investigational Review board.

**Fig. 1.**
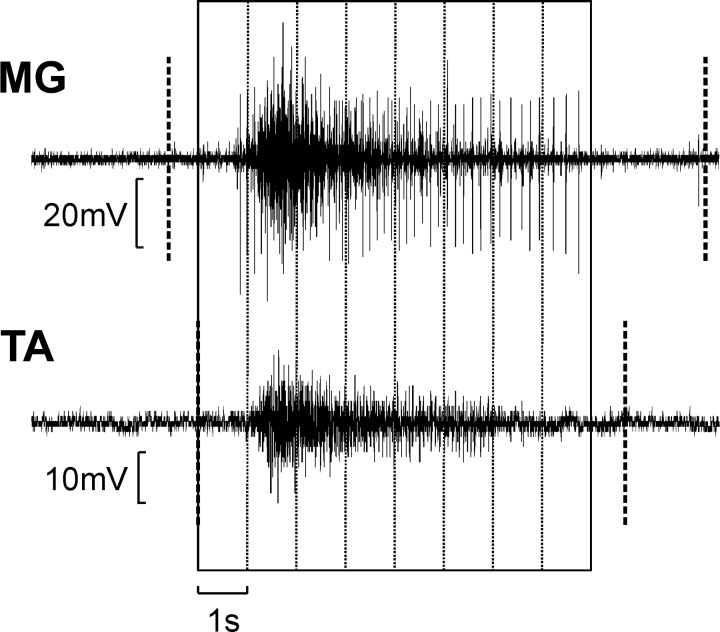
Method to calculate intermuscular coherence: example of electromyography (EMG) signals from one muscle pair (right medial gastrocnemius and tibialis anterior, MG-TA). First, the beginning and end of tonic spasms in both muscles were detected (thick dashed vertical lines). Second, data were selected only from periods where both muscles showed spasm activity simultaneously. Third, recordings were separated into 1.024-s-windows, ignoring any remaining spasm activity that could not fill a whole window. These windows were then used for the intermuscular coherence calculation.

EMG was measured from four muscles: right medial gastrocnemius (MG), tibialis anterior (TA), hamstrings (HM) and vastus lateralis (VL); these were chosen as agonist/antagonist pairs around the knee and ankle joints. Recordings were taken over a 24-h period for each participant using a data logger. This portable device was connected to surface electrodes (Superior Silver, no. 626SS; Uni-Patch, Wabasha, MN). The electrodes were trimmed (1 *×* 3 cm) and positioned in a pair over the distal third of the muscle (4-cm interelectrode spacing). EMG signals were amplified on the device (filter bandpass 30 Hz–1 kHz) and digitized with a 1-kHz sampling rate. In this way, we were able to record involuntary spasms that occurred while subjects went about their normal daily life activities. We first used the previously reported algorithm to mark periods in each muscle where tonic spasms occurred; full details are given in Winslow et al. (2015). Briefly, the algorithm calculated the integral of the rectified EMG in 10-ms windows and compared this with a threshold level computed from a baseline region without muscle activity. If at least five 10-ms-long windows in a 100-ms period exceeded the threshold, the algorithm detected a tonic spasm. This was then further classified as clonus if periodic bursts of EMG were detected with frequency between 4 and 12 Hz. Only tonic spasms without clonus were retained for further analysis. This is illustrated in Fig. 1, where the dotted vertical lines mark the detected onset and offset of the spams in each muscle shown. Note that the contraction onset was detected somewhat before the large burst of activity in each muscle; this reflects the early discharge of small motor units at the start of the spasm, which, though small, just rose above the detection threshold. Intermuscular coherence was calculated for all six possible combinations of muscle pairs. For a given muscle pair, coherence calculation used full-wave rectified EMG from periods in which both muscles had coincident spasms, with 1.024-s-long windows. These windows are shown in Fig. 1. Further details about the recording device and algorithm for classification of tonic spasms are described by Winslow et al. (2015).

Power spectra, coherence, and the significance limit for coherence were calculated via the methods described in our previous work (Baker et al. 2006), using a Fourier transform window size of 1,024 sample points, corresponding to a frequency resolution of 0.98 Hz. The number of windows used to calculate coherence varied from 271 to 6,808 across the different muscle pairs and subjects.

We took two approaches to assess the consistency of findings across subjects and muscle pairs. First, coherence spectra for a given muscle pair were averaged across all seven subjects; the significance limit (*P* < 0.05) for the average was calculated as described by Evans and Baker (2003). This provided a visual display of the average finding. Second, for an individual subject and muscle pair, we determined whether coherence was above the significance limit for a given frequency bin. The number of significant values was summed over all six muscle pairs and seven subjects, providing a maximum of 42 counts if significant coherence at that frequency was a universal finding. To assess significance on these plots, we used a binomial distribution to determine that a count of 6 would not be exceeded by chance more than 5% of the time, given that the probability of a single count was *P* = 0.05 on the null-hypothesis of no coherence.

## RESULTS

Across the subjects, the number of spasms in the 24-h recording period ranged from 286 to 1,714 [677 (479), mean (SD)]. The average spasm duration ranged from 3.8 to 23.5 s [9.6 (7.0) s].

**Fig. 2.**
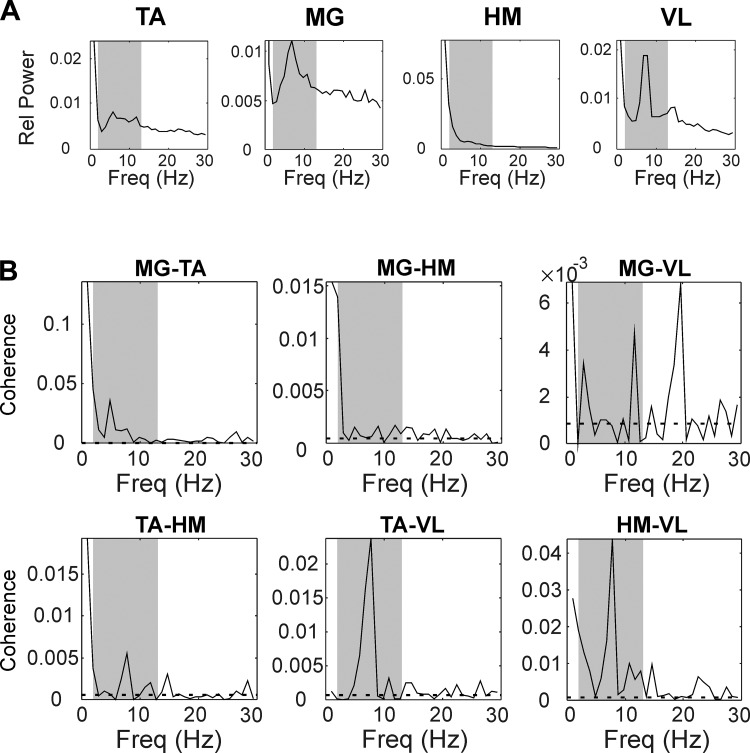
Results from a single subject, classified as American Spinal Cord Injury Association Impairment B. *A*: power spectra for the 4 recorded muscles, expressed as relative power (i.e., power divided by sum of power from 0 to 500 Hz). *B*: intermuscular coherence spectra for all 6 muscle pairs. Dashed horizontal lines represent significance limit (*P* < 0.05). In *A* and *B*, gray-shaded areas show frequency band at 2–13 Hz. TA, tibialis anterior; MG, right medial gastrocnemius; HM, hamstrings; VL, vastus lateralis; Freq, frequency.

Figure 2 shows coherence results from a single subject who was classified as ASIA A. Significant intermuscular coherence was observed at lower frequencies (2–13 Hz) for most muscle pairs (Fig. 2*B*). For some muscle pairs, peaks in coherence at low frequencies corresponded to peaks in power spectra of the two muscles analyzed (Fig. 2*A*).

**Fig. 3.**
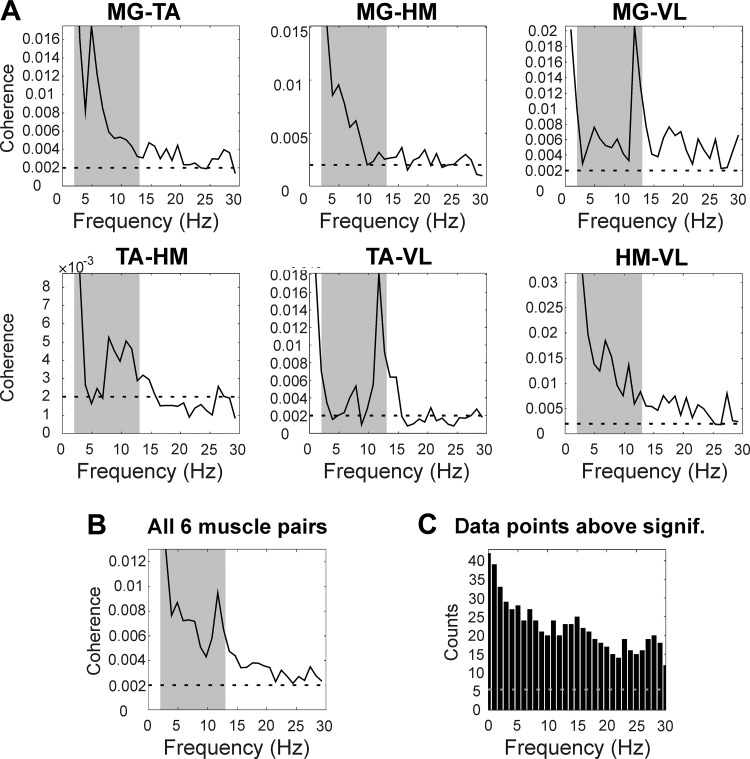
Average results. *A*: average coherence results from 7 spinal cord injury patients for all 6 muscle pairs. *B*: average coherence across 6 muscle pairs and 7 subjects. *C*: histogram showing the number of coherence measures above the significance limit summed over all 7 subjects and 6 muscle pairs. In *A* and *B*, gray-shaded areas show frequency band at 2–13 Hz. In *A–C*, dashed horizontal lines represent significance limit (*P* < 0.05).

Average results are presented in Fig. 3. Significant intermuscular coherence at low frequencies (2–13 Hz) was present in all six muscle pairs (Fig. 3*A*) and in the average across all muscle pairs (Fig. 3*B*). In some cases, peaks in coherence appeared on top of a decay in coherence values at low frequencies, which can be seen in average results from muscle pairs MG-TA and MG-HM (Fig. 3*A*). This provides confidence that there was a genuine oscillatory phenomenon, rather than just a non-oscillatory process containing a broad range of spectral frequencies. However, although coherence was clearly above the significance levels, it was considerably lower than we have previously reported in healthy subjects making voluntary contractions, where we often see coherence peaks above 0.05 (Jaiser et al. 2016).

Figure 3*C* shows the number of data points above significance across all subjects and all muscle pairs. Around half of all spectra available showed significant coherence up to 15 Hz, demonstrating that coupling at these frequencies was a robust finding.

Across subjects, there was no significant correlation between the coherence in the 2- to 13-Hz band (averaged across frequency bins and muscle pairs) and either spasm number over the 24-h recording period (*r*^2^ = 0.018) or mean spasm duration (*r*^2^ = 0.059; both *P* > 0.1). We were also interested in whether there would be consistent differences in coherence between subjects or muscle pairs. We therefore measured the mean coherence in the 2- to 13-Hz band for each spectrum available and carried out an ANOVA test with factors subject and muscle pair. Neither factor showed a significant effect (*P* > 0.1).

In all cases, we had a considerable quantity of data available for analysis, but this varied widely between muscle pairs and subjects (number of sections entering the Fourier transform ranged between 271 and 6,808). Coherence estimation is subject to a small bias, which depends weakly on the number of data sections available (Benignus 1969). We tested whether this could have affected our results by estimating the bias (equal to the reciprocal of the section number if coherence is low; Benignus, 1969) and computing the correlation with mean coherence in the 2- to 13-Hz band for each subject and muscle pair. There was no significant correlation (*r*^2^ = 0.0025, *P* > 0.1), indicating that the variable lengths of data available did not materially affect the results.

Finally, we assessed the consistency of the estimated coherence amplitude by splitting the available data into two and finding coherence in each half of the data separately. Across the six available muscle pairs and seven subjects, there was a significant correlation between mean 2- to 13-Hz coherence between the two halves of the data set, but this accounted for only a small fraction of the variance (*r*^2^ = 0.080, *P* = 0.001). This suggests that uncontrolled factors that changed between each half of the data set, e.g., time of day and posture, had more influence on the precise coherence amplitude than the muscle pair or subject.

**Fig. 4.**
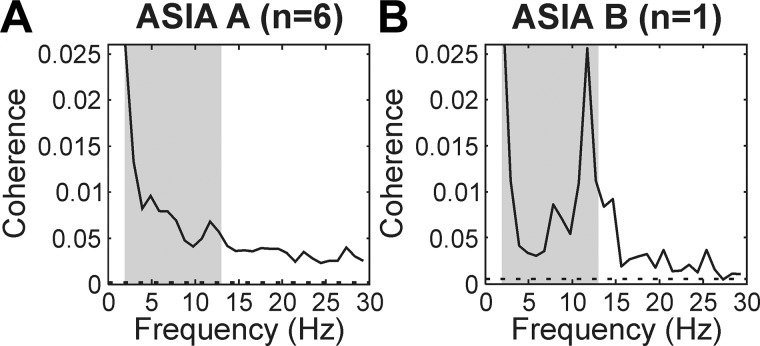
Separation of patients by American Spinal Cord Injury Association Impairment (ASIA) classification. Graphs show the intermuscular coherence averaged across 6 muscle pairs and all subjects with the same ASIA score. *A*: ASIA A (*n* = 6 subjects). *B*: ASIA B (*n* = 1 subject). Gray-shaded areas show frequency band at 2–13 Hz. Dashed horizontal lines represent significance limit (*P* < 0.05).

Our recordings were made mostly in subjects with ASIA score A, although a single subject was classified as ASIA B. It was of interest whether the results might be affected by the degree of impairment. Figure 4 plots coherence averaged across all six available muscle pairs, separately for the two groups of patients. It is clear that significant coherence in the 2- to 13-Hz band was a robust finding, visible in each subgroup. The single subject rated at ASIA B had a strong peak around 12 Hz (Fig. 4*B*), which was not so for the patients with ASIA A (Fig. 4*A*); however, it is difficult to draw conclusions from this single individual.

## DISCUSSION

Although intermuscular coherence in the beta-band (15–30 Hz) is well understood as having a predominantly cortical origin, the source of intermuscular coherence at lower frequencies is still under debate. In this study we demonstrated significant intermuscular coherence at low frequencies (2–13 Hz) during spontaneously occurring spasms in spinal cord injury patients. Our patients had a clinically complete injury to the motor system (ASIA A or B), suggesting a disconnected cord lacking supraspinal input. This therefore implies that spinal circuits are capable of producing intermuscular coherence in the range of 2–13 Hz.

Recent work has forced a reexamination of the concept of a “clinically complete” spinal cord lesion. As reviewed by Taccola et al. (2018), studies using electrical spinal stimulation in a total of 10 patients classified as ASIA A or B demonstrated a recovery of some voluntary contraction. This is likely to be due to axonal sprouting and regeneration induced by the stimulation, but it probably indicates that a limited number of surviving descending axons remained postinjury to form the substrate for stimulus-induced plasticity. Before stimulation was delivered, these axons were presumably insufficient to depolarize motoneurons to threshold, but they still may have provided subthreshold inputs. This is relevant to our study, because during a spasm motoneurons are active; weak common inputs from a descending pathway would then be capable of generating synchronization between motoneuron pools. In this regard, it is relevant to note that the data analyzed in this report were gathered around 4 years ago and first reported in Winslow et al. (2015). In the intervening period, some of the patients have shown a small recovery of function: three patients classified as ASIA A at the time of the recordings are now classed as ASIA B, and the single subject classified as ASIA B now meets the criteria for ASIA C on his most recent assessment. This might support the notion that, even at the time of the recordings, there was a very small residual descending drive, which has assumed more clinical significance with the passage of time.

We cannot completely exclude the possibility that the coherence we observed was related to residual descending pathways, but we believe it unlikely because the pattern of intermuscular coherence was very different in our recordings compared with that in healthy individuals. When uninjured controls perform a voluntary contraction, strong beta-band coupling is seen between muscle pairs in both the upper and lower limbs (Jaiser et al. 2016). This is typically larger than the coherence at lower frequencies. Following a selective experimental lesion of the corticospinal tract in monkey, beta-band intermuscular coherence is abolished, whereas weak intermuscular coherence at lower frequencies remains (Fisher et al. 2012).

One previous study used intermuscular coherence to analyze spasms in spinal cord injury. In a single paraplegic patient, Norton and colleagues (2004) found an isolated peak in intermuscular coherence at 16 Hz. Noting that this is a frequency typically associated with orthostatic tremor, these authors suggested that a spinal circuit may be responsible for the pathology of orthostatic tremor. Our results did not reveal a clear 16-Hz coherence peak, although peaks at slightly lower frequencies were seen for muscle pairs MG-VL and TA-VL (Fig. 3*A*). These may correspond to the previous finding. However, the appearance in only a limited subset of muscle pairs suggests that this was not the primary oscillatory mode generated by the spinal cord when descending control was reduced or absent. By contrast, coherence at lower frequencies (2–13 Hz) was a common observation.

The usual absence of corticomuscular synchronization at around 10 Hz suggests that intermuscular coherence around this frequency does not originate from the cortex (Boonstra et al. 2009b), and points instead to a subcortical origin. In healthy subjects, the brain stem is one possible candidate. We know that the reticular formation makes connections to a wide range of motoneurons innervating both upper and lower limb (Peterson 1979; Riddle et al. 2009). However, in the spinal cord injury subjects that we studied, brain stem descending systems were damaged, as well as the corticospinal tract. The only remaining possibility to generate these oscillations is thus the spinal cord itself, and its afferent feedback connections with the periphery.

Cremoux et al. (2017) measured cortico-muscular coherence in the upper limb in spinal cord injury subjects and showed that coherence was lower than in healthy subjects around 10 Hz. However, their published traces show relatively little coherence at or below 10 Hz. In addition, the level of the injury in these subjects ranged from C5 to T1, suggesting that some descending input would remain to the motoneuron pools of the elbow muscles that were recorded (innervated by segments C5–C7). It is therefore difficult to compare these findings with our own, where the lesion level (C3–C7) was substantially above the segmental innervation of the recorded leg muscles (L2–S2).

Intermuscular coherence at low frequencies has been observed in healthy adults during tasks involving bimanual coordination (5–12 Hz; de Vries et al. 2016), in-phase finger movements (~8 Hz; Evans and Baker 2003), bilateral precision grip (7–13 Hz; Boonstra et al. 2009b), walking (8–15 Hz; Halliday et al. 2003), and balance (6–11 Hz; Boonstra et al. 2009a). We speculate that these tasks could involve similar spinal circuitry to that recruited during spasms after spinal cord injury. Interestingly, bilateral synchronization is a common finding in the literature on this frequency band. The spinal cord is known to contain populations of commissural interneurons, which can mediate coupling between the two sides (Bannatyne et al. 2003; Jankowska et al. 2009). These also were recently demonstrated in primate cervical cord (Soteropoulos et al. 2013), so a spinal contribution to bilateral synchronization at low frequencies is plausible even for tasks involving bimanual action.

Intermuscular coherence at 3–10 Hz has also been reported in myoclonus dystonia, which is a movement disorder with a genetic basis (Foncke et al. 2007; Grosse et al. 2004; van der Meer et al. 2010). Similar to the spasms investigated in the present study, this is characterized by involuntary muscle contractions. These tend to be prolonged and cause twisting movements, possibly accompanied by myoclonic jerks (Grosse et al. 2004). Intermuscular coherence at low frequencies in these patients correlates with the presence of dystonia (Foncke et al. 2007). Low frequency intermuscular coherence is also of greater amplitude in subjects with writer’s cramp (a focal task-specific dystonia) than in healthy controls (Choudhury et al. 2018). Dystonia is believed to occur because of basal ganglia dysfunction, which results in decreased cortical inhibition (Morgante and Klein 2013). This would alter descending input to the cord, possibly providing a common pathway for the generation of this pathological activity with spinal cord injury.

We have used intermuscular coherence, which conveniently measures shared input to muscle pairs. By contrast, Bravo-Esteban et al. (2014, 2017) measured intramuscular coherence using paired recordings from the TA muscle. In spinal cord-injured subjects with incomplete lesions (ASIA C or D), they found significant coherence at lower frequencies (10–16 Hz) during brief voluntary contractions. Interestingly, the magnitude of this coherence was positively correlated with measures of spasticity. It is possible that the same subcortical circuit could generate these oscillations in healthy individuals and in those with both complete and incomplete spinal cord injuries. For incomplete injuries, this circuit may be under reduced levels of descending control, leading to spasticity as classically defined (Lance 1980). When the injury is complete, the total release from descending influences could produce the overt spasms that we observed.

One unanswered question is the extent to which low-frequency oscillations can be generated solely by the isolated spinal cord, or whether they require intact sensory input. In myoclonus dystonia, van der Meer and colleagues (2010) used external perturbations to explore the role of sensory feedback and concluded that it probably played a minimal role in low-frequency drive. Previous work from this laboratory showed that sensory input to the cord is configured to produce phase cancellation around these frequencies with descending input from the cortex (Koželj and Baker 2014). This suggests the possibility of a subtle interplay between central and peripheral circuits that can either cancel, or possibly generate, low-frequency activity. This would be similar to the situation for the spinal central pattern generator circuits that operate at much lower frequencies to generate locomotor activity. Although these can generate rhythmic alternation of activity in an isolated spinal cord (“fictive locomotion”), in the intact animal sensory input is integrated into the rhythm generation, allowing for example adjustment of walking speed to match the speed of a treadmill (Frigon 2017).

The present study examined tonic spasms, which are not accompanied by overt limb oscillations. Another important type of spasm after spinal cord injury is clonus, in which a limb segment generates rhythmic contractions. These are typically in the same 2- to 13-Hz frequency range as examined in the present study (Winslow et al. 2015). Early reports proposed that clonus was the result of feedback oscillations in the stretch reflex loop (Hagbarth et al. 1975; Hidler and Rymer 1999). Later work (Beres-Jones et al. 2003) demonstrated that antagonist muscle pairs could show in-phase activity, which would not be expected from stretch-evoked activity because one muscle would be slack when the other was stretched. Additionally, some bouts of clonus could occur without accompanying oscillations in muscle length. This led to the suggestion that clonus was produced by a central, probably spinal oscillator, which could be modulated by sensory input. It seems very likely that the oscillations that we have observed during tonic spasms and the overt contractions of clonus are manifestations of the same underlying neural process, but at different ends of the possible amplitude range.

Although the greatest coherence that we observed was in the band at 2–13 Hz, in many cases significant coherence was also seen for higher frequencies (Fig. 2–4). The presence of significant beta coherence after complete spinal cord injury appears to contradict the idea that coherence in this band arises from the corticospinal tract. However, although it was significantly different from zero, the beta-band coherence in the present data was very low, around 0.003 in the average of Fig. 3*B*. This is to be compared with average values around 0.03 in the results from healthy control subjects presented by Fisher et al. (2012). Indeed, in that work, the significance limit for the coherence spectra was often around 0.01, reflecting the shorter duration recordings available compared with our extensive data gathered over 24 h. Coherence at the level that we observed would therefore not have been detectable with the use of more usual recording durations. It appears that subcortical circuits may be capable of generating beta-band intermuscular coherence but that this is an order of magnitude lower than that generated by descending corticospinal drive.

In summary, this is the first report to demonstrate that intermuscular coherence at low frequencies (2–13 Hz) can be produced by spinal circuits in a situation where descending drive is abolished or severely reduced. Although we cannot rule out a contribution from supraspinal structures in the intact nervous system, it is likely that at least part of the synchronous activity in this frequency range observed in healthy subjects also has a spinal origin.

## GRANTS

This work was supported by National Council for Scientific and Technical Development, Brazil Grant 205440/2014-2, Wellcome Trust Grant 101002/Z/13/Z, National Institute of Neurological Disorders and Stroke Grant NS-30226, and The Miami Project to Cure Paralysis.

## DISCLOSURES

No conflicts of interest, financial or otherwise, are declared by the authors.

## AUTHOR CONTRIBUTIONS

C.T. conceived and designed research; K.G., J.B., and C.T. performed experiments; S.A.A., S.N.B., K.G., and J.B. analyzed data; S.A.A., S.N.B., and C.T. interpreted results of experiments; S.A.A. prepared figures; S.A.A. drafted manuscript; S.A.A., S.N.B., and C.T. edited and revised manuscript; S.A.A., S.N.B., K.G., and J.B. approved final version of manuscript.
